# Supplementary material for: Towards non-blind optical tweezing by finding 3D refractive index changes through off-focus interferometric tracking
Source: Nat Commun. 2021 Nov 26;12:6922. doi: 10.1038/s41467-021-27262-z (PMC8626468; doi:10.1038/s41467-021-27262-z)
Supplement: Supplementary file 2 — Description of Additional Supplementary Files [file 41467_2021_27262_MOESM2_ESM.docx]

**Description of Additional Supplementary Files:**

**Supplementary Movie 1:** Rotation of a single B-cell (10µm in diameter) perpendicular to the optical axis by 2 blind optical tweezers

**Supplementary Movie 2:** Rotation of a dividing B-cell (each 10µm in diameter) perpendicular to the optical axis by 2 blind optical tweezers

**Supplementary Movie 3:** Unstable rotation of a 70µm cancer cell cluster perpendicular to the optical axis by 3 blind optical tweezers

**Supplementary Movie 4:** Off-focus total intensity and off-focus interference intensity of beam no. 6 for different particle positions.

**Supplementary Movie 5:** Stable optical lifting and holding of a 170µm cancer cell cluster with 8 optical traps of each 56 mW laser power

**Supplementary Movie 6:** Individual beads, i.e. each local refractive index changes (displayed in pseudocolors) in the center of a bead cluster can be reproduced by the OFI beam scans.
